# Safe Farming: Ultrafine Bubble Water Reduces Insect Infestation and Improves Melon Yield and Quality

**DOI:** 10.3390/plants13040537

**Published:** 2024-02-16

**Authors:** Jo-Chi Hung, Ning-Juan Li, Ching-Yen Peng, Ching-Chieh Yang, Swee-Suak Ko

**Affiliations:** 1 Agricultural Biotechnology Research Center, Academia Sinica, Taipei 115, Taiwan; rochehorng@gmail.com; 2 Biotechnology Center in Southern Taiwan, Academia Sinica, Tainan 711, Taiwan; 3 Season Agricultural Technology Co., Ltd., Tainan 711, Taiwan; ningjuan.farm@gmail.com (N.-J.L.); pchingyen@gmail.com (C.-Y.P.)

**Keywords:** jasmonate, muskmelon, nanobubbles water, pest damage, trichome, yield

## Abstract

Melon pest management relies on the excessive application of pesticides. Reducing pesticide spraying has become a global issue for environmental sustainability and human health. Therefore, developing a new cropping system that is sustainable and eco-friendly is important. This study found that melon seedlings irrigated with ultrafine water containing H_2_ and O_2_ (UFW) produced more root hairs, increased shoot height, and produced more flowers than the control irrigated with reverse osmosis (RO) water. Surprisingly, we also discovered that UFW irrigation significantly reduced aphid infestation in melons. Based on cryo-scanning electron microscope (cryo-SEM) observations, UFW treatment enhanced trichome development and prevented aphid infestation. To investigate whether it was H_2_ or O_2_ that helped to deter insect infestation, we prepared UF water enrichment of H_2_ (UF+H_2_) and O_2_ (UF+O_2_) separately and irrigated melons. Cryo-SEM results indicated that both UF+H_2_ and UF+O_2_ can increase the density of trichomes in melon leaves and petioles. RT-qPCR showed that UF+H_2_ significantly increased the gene expression level of the trichome-related gene *GLABRA2* (*GL2*). We planted melons in a plastic greenhouse and irrigated them with ultrafine water enrichment of hydrogen (UF+H_2_) and oxygen (UF+O_2_). The SPAD value, photosynthetic parameters, root weight, fruit weight, and fruit sweetness were all better than the control without ultrafine water irrigation. UFW significantly increased trichome development, enhanced insect resistance, and improved fruit traits. This system thus provides useful water management for pest control and sustainable agricultural production.

## 1. Introduction

Ultrafine bubble water (UFW), also known as nanobubble or microbubble water, contains small molecules less than 100 nm in diameter which can carry gases on their surfaces [1]. UFW water penetrates the soil quickly and can be taken up by roots more effectively, enhancing plant growth and development. It has been widely used in crop production [2,3]. It has been reported that plants irrigated with UFW have increased seed germination [4,5,6], show significantly enhanced rooting and adventitious root development [7], and enhanced root nutrient uptake and increased nutrient use efficiency [8]. Many reports have shown UFW irrigation increased crops yield and quality, such as in rice [9], lettuce [10], tomatoes [11,12], cucumbers [13], muskmelon [14], and strawberries [15].

Ultrafine water enriched with hydrogen prolongs the vase life and the quality of cut flowers [16,17]. Also, it extends the shelf life of kiwifruit [18] and strawberries [19]. It also plays an essential role in plant tolerance to abiotic stresses. Hydrogen pretreatment can improve the salt stress resistance of rice and *Arabidopsis* [20]. It has been reported that molecular hydrogen (H_2_) has antioxidant activity, removing reactive oxygen species (ROS) and reactive nitrogen species (RNS) and reducing free radical toxicity [21,22].

Melon or muskmelon (*Cucumis melo* L.) is a popular fruit consumed worldwide, and it has a significant economic value in the global market. Melon crops are susceptible to infestation by a variety of insects, such as aphids, thrips, whiteflies, cucumber beetles, and spider mites [23]. Aphids are tiny insects that suck the sap from the plant and can cause stunted growth, curled leaves, transmit viruses, and decrease crop yield. Pathogens such as aphid-transmitted melon cucumber mosaic virus (CMV) and watermelon mosaic virus-2 (WMV-2) [24] cause severe damage to melon plants and lead to reduced yields and decreased fruit quality. Consequently, farmers spray pesticides frequently, which causes a food safety issue. Therefore, the development of a new cropping system for the Sustainability Assessment of Farming and the Environment (SAFE) [25] in melon production is very important.

Trichomes are hair-like outgrowth on the surface of plant organs such as leaves, stems, and flowers. Trichomes act as a physical barrier against herbivorous insects by deterring their ability to feed on the plant and reducing insect movement. Plants with higher trichome density are known to be more resistant to insects [26] and have a strong positive correlation between trichome density and insect resistance [27]. Moreover, glandular trichomes can also produce volatile compounds that are toxic or repellent to insects [28]. Jasmonic acid (JA) is a herbivory-induced hormone that participates in terpene biosynthesis [29]. Methyl jasmonate (Me-JA) treatment significantly enhanced the expression of several monoterpene and sesquiterpene synthases. Research showed that the knockout of an HD-ZIP IV transcription factor (TF), woolly (wo), led to a significant defect in trichomes and a reduction of terpene levels and is associated with insect resistance in tomatoes [30]. Me-JA induced type VI glandular trichome formation on the newly expanding tomato leaves, thus decreasing herbivore insect populations [31]. The gene regulation network controlling trichome development is complex [32]. It is regulated by GLABRA1 (GL1), GLABRA2 (GL2), GLABRA3 (GL3), and TRANSPARENT TESTA GLABRA1 (TTG). The loss of function of these TFs showed glabrous phenotypes [33,34,35,36,37,38,39]. GLABRA3 (GL3) is a wound-induced trichome formation acting downstream of the JA signaling pathway [35]. TRIPTYCHON (TRY) is a negative regulator of trichome and root hair development [40]. It has been reported that JAZ is required for jasmonate-meditated glandular trichome development in *Nicotiana benthamiana* [41] and rice [42].

The application of hydrogen in agriculture has attracted much attention over the last decade and has several prospects [43]. However, to the best of our knowledge, there is no report on UFW-induced JA and increasing trichome development. The aims of the present work were as follows: (i) to observe whether UFW improves melon seedling growth and fruit production; (ii) to understand whether UFW enhances pest resistance; and (iii) to know whether UFW affects the JA-pathway and induces trichome development in melons.

## 2. Results

### 2.1. UFW Treatment Improved the Growth of Melon Seedlings

**Figure 1:**
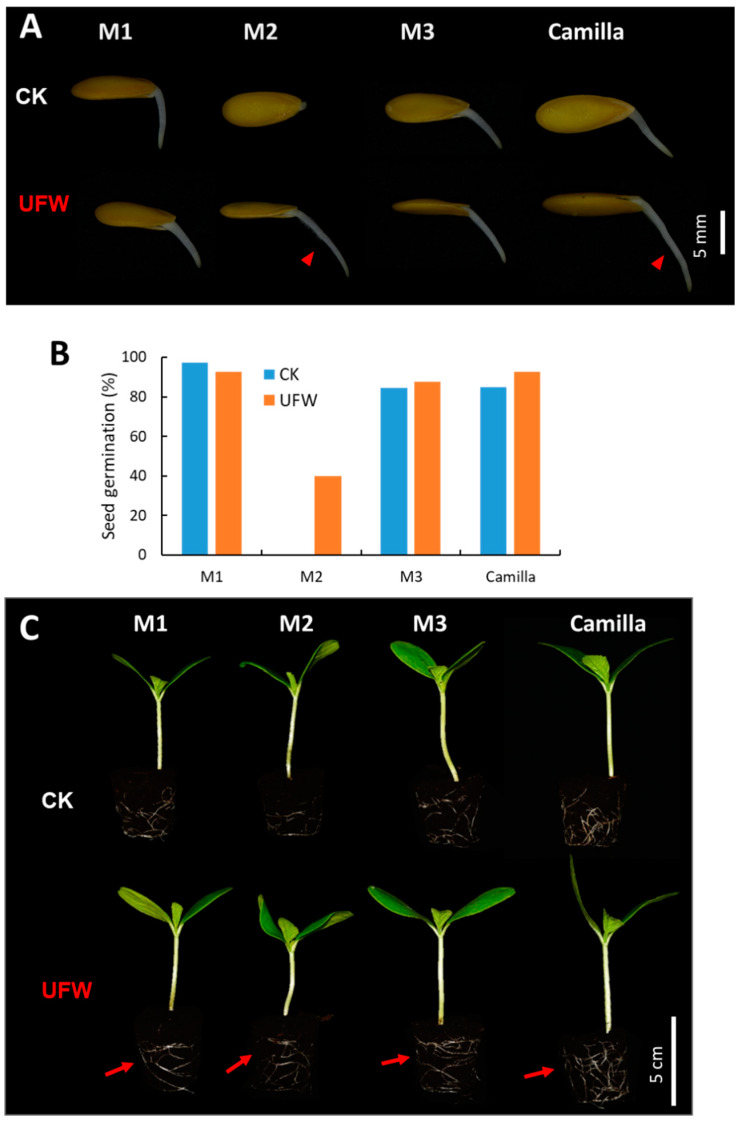
Ultrafine water affected seed germination and rooting of melons. (**A**) Effects of ultrafine water (UFW) on melon seed germination. Four melon varieties, each with 40 seeds, were germinated in Petri dishes containing RO water and UFW. Arrows show the presence of root hairs on the root at 1 day after seed germination. (**B**) Germination rate of melon seeds at 7 days after germination. (**C**) Melon seedlings grown in plug trays containing peat moss at 7 days after sowing (DAS). Arrows show vigorous root development.

To understand the effects of ultrafine water (UFW) on seed germination and seedling growth, we tested four lines of melon seeds: M1, M2, M3, and Camilla. Forty seeds per line were imbibed in UFW and RO water overnight, and then placed in square Petri dishes containing UFW and reverse osmosis (RO) water as a control (CK), respectively. The dishes were then placed in a growth chamber in the dark and set to a constant temperature of 28 °C. After germination for one day, the melon seeds in UFW produced longer and more root hairs than CK (Figure 1A). The germination rates of UFW-treated seeds of M2, M3, and Camilla were higher than those of CK (Figure 1B). We transplanted the germinated melon seeds into a #104 plug tray filled with peat moss and raised the seedlings in the greenhouse. Treatment with UFW produced more vigorous roots and seedlings than CK at 7 days after transplantation (Figure 1C).

### 2.2. UFW Reduced Aphid Infestation of Seedlings

**Figure 2:**
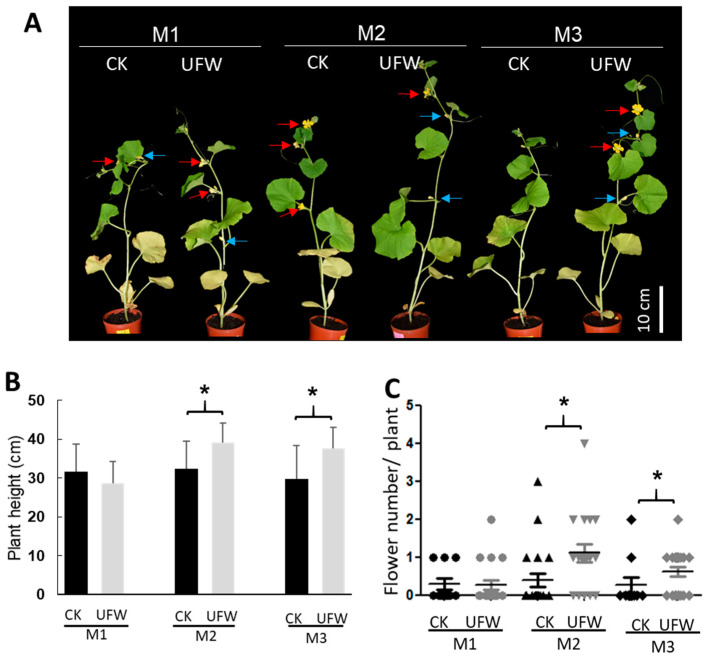
UFW irrigation affected the growth of melon seedlings. (**A**) The phenotype of melon potted plants at 14 days after transplantation (DAT). Red arrows indicate the fresh flowers, blue arrows indicate the wilting flowers. Bars, 10 cm. (**B**) Plant height of melons. Error bars represent the standard error of the mean (*n* = 10–21 per treatment). (**C**) Scatter plot of flower number per plant at 14 DAT. Horizontal lines indicate mean values (*n* = 10–21). *, significant differences between CK and UFW treatment were determined using Student’s *t*-test at *p* < 0.05 (**B**,**C**).

In order to understand the effect of UFW on the growth of melon seedlings, we transplanted melon seedlings from plugs into pots and placed them in the same growth chamber to grow, but irrigated them with RO water and UFW. At 14 days after transplanting, the plant heights of the UFW-irrigated melon lines M2 and M3 were higher and produced more flowers than those of CK (Figure 2).

**Figure 3:**
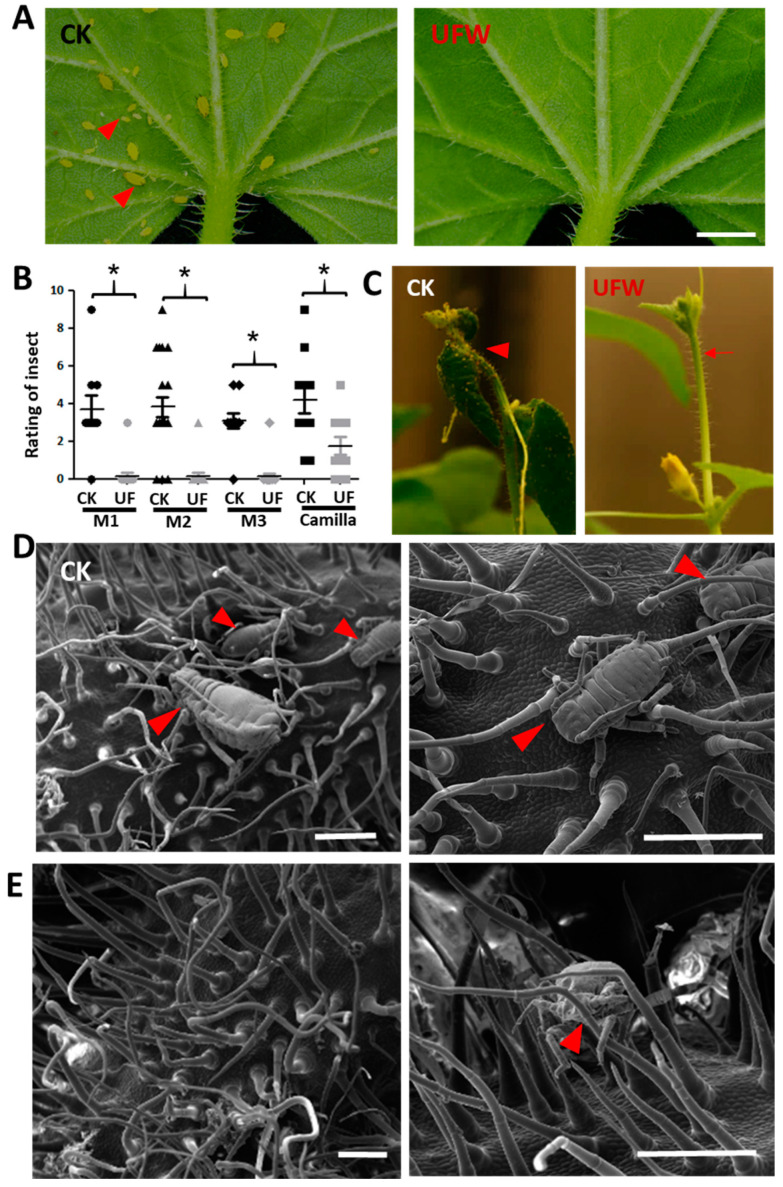
UFW irrigation affected aphid infestation on melon seedlings. (**A**) Phenotype of melon leaves attacked by aphids 14 days after transplantation. Scale bars, 2 mm. (**B**) Scatter plot of aphid infestation rating. A rating of 0 indicates no aphids were observed, and 9 indicates a high aphid density. Horizontal lines indicate mean values (*n* = 10–21). *, significant differences between CK and UFW treatment were determined using Student’s *t*-test at *p* < 0.05. (**C**) Aphids attacked the young flower buds of melon (arrowhead). Trichomes development after UFW treatment (arrow). (**D**) Cryo-SEM showed aphid infestation on flower buds of CK (**D**) and UFW (**E**). The arrowheads point to the aphids. Scale bars, 500 µm (**D**,**E**).

We found that aphids attacked melon seedlings in the growth chamber 14 days after transplantation (DAT). Surprisingly, it was found that the aphid density in the leaves and flower buds of melons irrigated with UFW was lower than that of the control group (Figure 3A–C). We performed cryo-SEM and observed that the trichomes of CK were fewer and drooping, and the mouthparts of aphids could easily reach the leaf surface (Figure 3D). However, the UFW-irrigated melon plants have upright and dense trichomes that interfered with aphid movement and feeding (Figure 3E).

### 2.3. Effect of Hydrogen-Rich (UF+H_2_) or Oxygen-Rich (UF+O_2_) Ultrafine Water on Trichome Development

**Figure 4:**
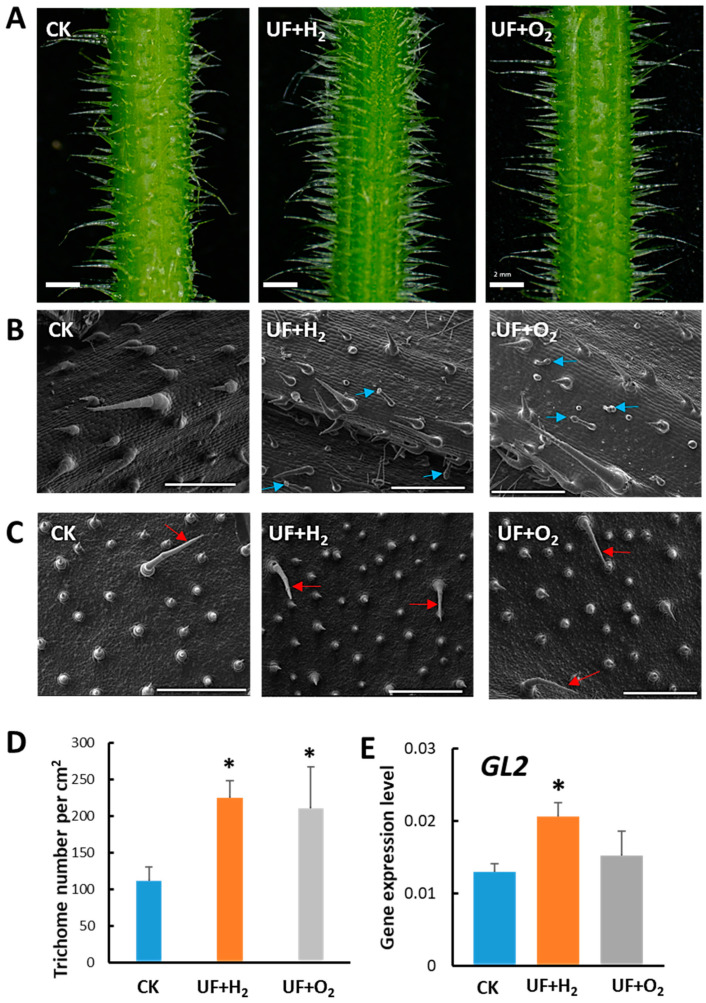
Hydrogen-rich or oxygen-rich ultrafine water irrigation affected the development of trichomes in melon cv. “Camilla”. (**A**) Dissecting microscope observation of the development of trichomes in melon petioles after irrigation with ultrafine water enrichment of hydrogen (UF+H_2_), oxygen (UF+O_2_), and RO water (Ck), respectively. Bars, 2 mm. (**B**) Cryo-scanning electron microscope (cryo-SEM) showing trichomes on the midribs of the melons. Blue arrows indicate the presence of granular trichomes. Bars, 500 µm. (**C**) Cryo-SEM showed the development of trichomes on the abaxial of newly established young leaves of melon (red arrows). Bars, 500 µm. (**D**) Trichome density in melon petioles irrigated with RO water, UF+H_2_, and UF+O_2_, *n* = 3 to 6. (**E**) RT-qPCR showed *GLABRA2* (*GL2*) gene expression patterns in young melon leaves irrigated with UF+H_2_, UF+O_2_, and RO water control (CK). *, significant differences between CK and UFW treatment were determined using Student’s *t*-test at *p* < 0.05 (**D**,**E**).

Our previous experiments showed that UFW containing both H_2_ and O_2_ significantly increased trichome density and deterred aphid infestation (Figure 3). We were, thus, interested to know whether this phenomenon was due to the effect of H_2_ or O_2_ molecules. Hence, we prepared the UF water enrichment of pure hydrogen (UF+H_2_), pure oxygen (UF+O_2_), and RO water (CK), and irrigated melon cultivar “Camilla”, respectively. Melon plants irrigated with UF+H_2_ produced taller and denser trichomes on the petioles, leaf veins, leaves, and leaf tip compared with CK. Meanwhile, UF+O_2_ irrigated plants had longer trichomes than CK (Figure S1). Under a dissecting microscope, we could observe that melons irrigated with UF+H_2_ produced longer and denser trichomes on the mid-rib than those irrigated with UF+O_2_ or CK. We took pictures and measured trichome density in petioles and found that UF+H_2_ and UF+O_2_ irrigation significantly increased trichome density (Figure 4A,D).

Under a cryo-SEM microscope, we observed that long unicellular trichomes and granular trichomes were produced in the midrib of melon leaves after UF+H_2_ or UF+O_2_ irrigation. However, at the study stage, there were no glandular trichomes in the midrib of CK (Figure 4B). Compared with CK, the morphology of midrib glandular trichomes in melon leaves irrigated with UF+H_2_ or UF+O_2_ were multicellular with medium and long stalks and small globular secretory heads (Figure 4B, blue arrows). The abaxial leaves had a denser trichome density than CK after UF+H_2_ and UF+O_2_ irrigation (Figure 4C,D). Consistently, we performed RT-qPCR and found that the positive regulator of the trichome development marker gene, *GLABRA2* (*GL2*), was significantly increased in the premature young leaf after irrigation with UF+H_2_ (Figure 4E).

### 2.4. Enrichment of Hydrogen-Induced Jasmonic Acid Accumulation

**Figure 5:**
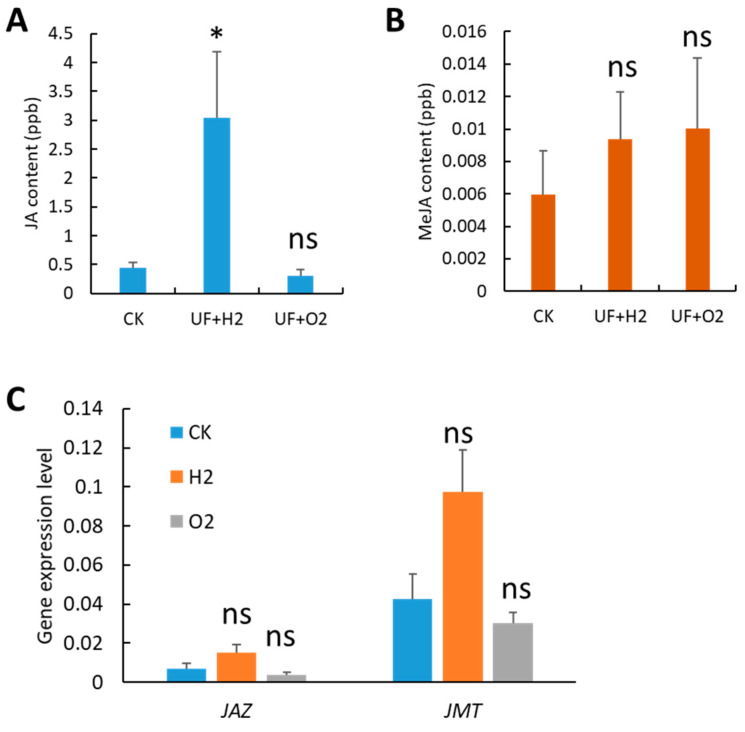
Melon irrigated with hydrogen- and oxygen-rich ultrafine water altered jasmonic acid (JA) and methyl-JA (MeJA) contents, and gene expression patterns. (**A**) JA content. (**B**) MeJA content. (**C**) The gene expression level of *JASMONATE ZIM DOMAIN PROTEIN* (*JAZ*) and *JA carboxyl methyltransferase* (*JMT*). The gene expression level was normalized to two housekeeping genes: *Actin* (MELO3C023264) and *ADP ribosylation factor 1* (*ADP*, MELO3C023630). Error bars represent the standard error of the mean (*n* = 3). Student’s *t*-test was used to find the significant difference between CK and UF+H_2_ or UF+O_2_ treatment. *, *p* < 0.05; ns, not significant.

We detected the JA and MeJA contents in melon leaves and found that JA was significantly increased (6.9-fold) under UF+H_2_ treatment compared to the CK (Figure 5A). Although UF+H_2_ and UF+O_2_ slightly increased the MeJA content, there was no statistically significant difference at *p* < 0.05 (Figure 5B). Our RT-qPCR showed that *JASMONATE ZIM DOMAIN PROTEIN* (*JAZ*) and *JA carboxyl methyltransferase* (*JMT*) were upregulated after UF+H_2_ treatment but there was no statistically significant difference at *p* < 0.05 (Figure 5C).

### 2.5. Effect of UF Water on Photosynthesis Parameters, Fruit Yield and Quality

**Figure 6:**
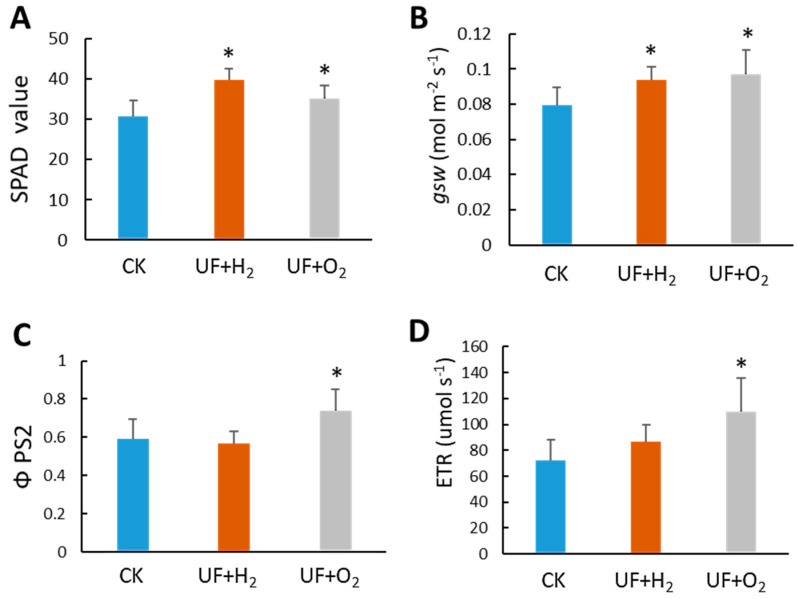
Ultrafine water irrigation affected the photosynthesis capacity of melons. (**A**) Chlorophyll content in melons. The SPAD value was measured on the 4th leaf at the late stage of fruit maturity. *n* = 4 plants. The Li600 Porometer/Fluorometers meter detected the photosynthesis parameters of (**B**) stomatal conductance (gsw); (**C**) ΦPSII, the quantum yield of PSII calculated from fluorescence; and (**D**) the electron transport rate (ETR) of L1 melon leaves. Student’s *t*-test was used to find the significant difference between UFW and the regular tap water (CK). *, *p* < 0.05. Error bars represent the standard error of the mean (*n* = 4).

We grew melons in a greenhouse to understand the effect of UF+H_2_ and UF+O_2_ irrigation on melon fruit production. All crop management practices were similar except for irrigation water use. We measured chlorophyll content and photosynthesis parameters using a SPAD meter and Li-600 Porometer/Fluorometers meter, respectively. The results showed that H_2_ and O_2_ enrichment significantly increased SPAD values (representing chlorophyll content) and stomatal conductance (gsw). UF+O_2_ increased the quantum yield of PSII calculated from fluorescence (ΦPSII) and electron transport rate (ETR) (Figure 6).

**Figure 7:**
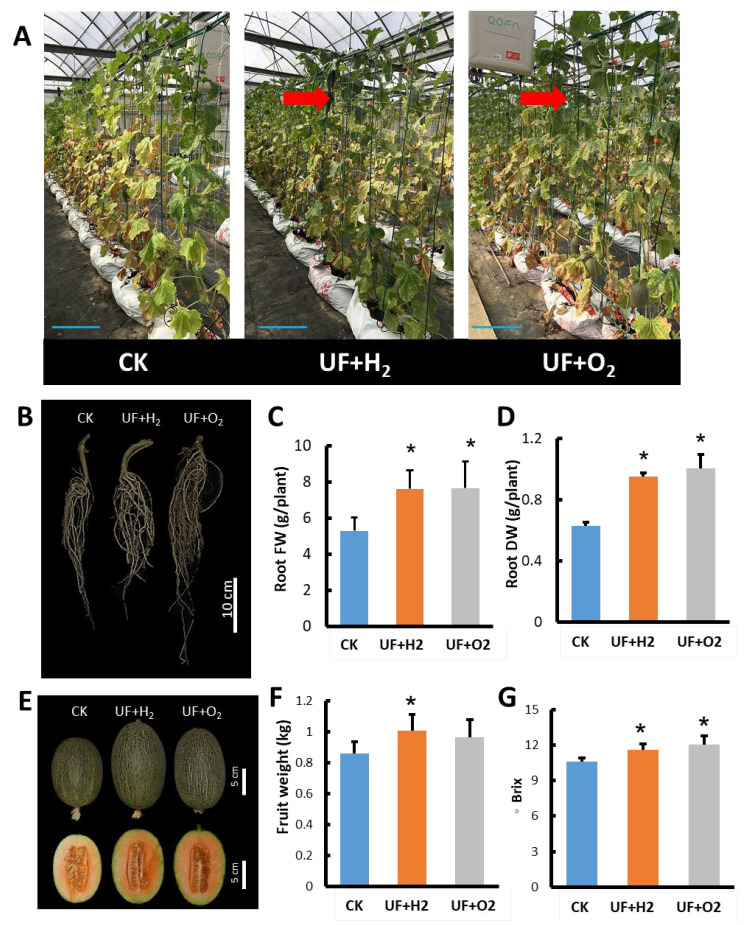
UFW irrigation affected fruit weight and sweetness of melon cv. “Camilla”. (**A**) Melons were planted in a greenhouse. Photo taken 42 days after pollination. Bars, 20 cm. (**B**) Root morphology at harvest stage. (**C**) Root fresh weight of each plant. (**D**) Root dry weight per plant. (**E**) Melon fruits at 5 days after harvest. (**F**) Average fruit weight of melon. (**G**) The sweetness of melon fruits. UF+H_2_, hydrogen-rich ultrafine water irrigation. UF+O_2_, oxygen-rich ultrafine water irrigation. CK, irrigated with tap water. Bars, standard deviation of 22 plants. Student’s *t*-test was used to find significant difference between CK and UF+H_2_ or UF+O_2_ treatment. *, *p* < 0.05.

During the growth process of melons in the late harvest stage, melon plants irrigated with UF+H_2_ or UF+O_2_ retained more green leaves (Figure 7A, arrows) than CK irrigated with tap water. After irrigation with UFW, the root system of the melon plants developed more vigorously, and the fresh weight and dry weight of the roots were significantly higher than the control (Figure 7B–D). UF+H_2_ irrigation increased the melon fruit size and weight (Figure 7E,F). Furthermore, UF+H_2_ and UF+O_2_ irrigation were both able to increase melon fruit sweetness (Figure 7G).

## 3. Discussion

This study showed that UFW irrigation significantly improved melon seed germination, seedling growth, and enhanced root development. Our greenhouse experiment also showed that the UFW enrichment of hydrogen (UF+H_2_) or oxygen (UF+O_2_) produced higher root biomass than the control without UFW treatment. The robust root system contributed to plant growth and development. Previous studies have demonstrated that hydrogen-rich water increased auxin and GA3 biosynthesis and enhanced root development [44]. It regulated heme oxygenase-1/carbon monoxide pathways and increased root development [13]. Some researchers have suggested that hydrogen has antioxidant properties, which can help to reduce oxidative stress in plants, improve nutrient uptake by plants, and improve overall plant growth and development [6,8,10,13,22]. Hydrogen molecules are not easy to apply. Nonetheless, water electrolysis produces hydrogen gas, which is easily fused into ultrafine water and provides a good solution for agricultural applications.

Our data indicated that UFW positively affects crop production compared to the previous reports on cucumber [45] and maize [46]. This study showed that hydrogen enrichment water is better than oxygen. Compared to H_2_-rich water, studies of O_2_-rich water on plant growth are rare. Recently, a report highlighted that the nanobubble water enrichment of O_2_ improves soil structure and microbial diversity, thereby increasing tomato yield [47]. The UFW enrichment of O_2_ could enhance oxygen delivery to soil and promote aerobic respiration [48]. Some reports indicated that high O_2_ content in UFW does not necessarily lead to better crop performance. In a previous study on maize treated with dissolved oxygen (DO) concentrations of 10, 20, and 30 mg/L, a moderate DO concentration of 20 mg/L had the highest root growth and yield [46]. In this study, we grew melons in a well-ventilated soilless medium of peat moss, which may reduce the positive effects of UFW. A more significant impact would be expected if the UFW irrigated a high-density clay field with poor aeration. It is hypothesized that UF+O_2_ may benefit plant survival under flooding-induced hypoxic conditions.

We observed that melons irrigated with UF+H_2_ or UF+O_2_ retained green leaves, and in the later stages of melon development, the leaves contained higher chlorophyll. This is a beneficial trait that can increase the rate of photosynthesis and produce more assimilates for fruit development, thereby increasing the fruit weight and sweetness of the melon (Figure 7). As reported previously, hydrogen enrichment water increased strawberry fruit flavor and quality [15].

Non-glandular trichomes have been reported to play a role in the mechanical defense against insects, while glandular trichomes secrete metabolites such as terpene [49]. In this study, glandular trichomes were found in the midrib of melon leaves after irrigation with UFW (UF+H_2_ and UF+O_2_) but not in the control (Figure 4). To our knowledge, this is the first report showing that ultrafine water can increase trichome density and induce glandular trichome development. We discovered that UF+H_2_ can induce JA-biosynthesis genes and enhance root and trichome development. Trichomes deter herbivores and reduce insect damage. In the future, it will be worth investigating what secondary metabolites were induced after UFW treatment.

JA is known to be involved in trichome development [30,31]. In this study, we found that a trichome initiation marker gene *GL2* was significantly upregulated in young leaves after UF+H_2_ treatment (Figure 4E), further supporting the notion that hydrogen may induce JA and enhance trichome initiation to prevent herbivory infestation and improve plant growth. Our data show that irrigating melon with UFW (H_2_ and O_2_) improved the resistance of melons to aphid infestation (Figure 3). Furthermore, we found that the UFW enrichment of H_2_ plays an important role in trichome development due to the upregulation of JA biosynthesis genes and increased JA accumulation in the plants irrigated with the UFW enrichment of H_2_ (Figure 5). This enhanced trichome formation and deterred insects or interfered with their feeding and growth, making the plants less susceptible to damage. Furthermore, reducing pest infestation will reduce systemic viral infection problems. Overall, these data indicate that hydrogen-rich water upregulates JA-pathway marker genes and increases melon trichome development, supported by the upregulation of *GL2* transcripts. A high density of globular trichomes may help to resist aphids. In the future, it will be worth conducting more extensive research on the underlying mechanisms through which UFW induces JA, enhances trichome development, and confers insect resistance.

We demonstrated that UFW increased trichome density and prevented aphid feeding (Figure 3 and Figure 4), induced more flower development, increased fruit weight, and increased the sweetness of melon fruits (Figure 7). All these beneficial effects contribute to melon crop production. These results demonstrate that hydrogen-rich water management has excellent potential as a natural, non-toxic pest and disease control treatment in crop plants. This might reduce pesticide spraying and improve food security. The UFW-induced JA response facilitates the establishment of a natural defense system in crop plants against insect attacks. Therefore, agriculture is safe without relying on pesticides. It is an environmentally friendly agricultural practice that increases crop yields and fruit quality and reduces pest damage. In addition, UFW has hydrophobic and surface charge properties that enhance the release and absorption of soil nutrients, thereby reducing fertilizer demand [9]. This will reduce the carbon footprint in crop production and enable sustainable agricultural production. Some studies emphasize enhancing insect resistance through genetic engineering trichome genes, but due to consumer concern about the biosafety of the genetically modified organisms (GMO), here we proposed a hydrogen-rich UFW irrigation method which would be more acceptable to consumers as it does not include GMO. Furthermore, hydrogen-rich water is safe and easy to use [50].

## 4. Materials and Methods

### 4.1. Ultrafine Water Preparation

We used a Hydrogen–Oxygen Ultrafine bubble system model HOU-3 (Season Agricultural Technology Co., Ltd., Tainan, Taiwan) to prepare ultrafine bubble water (UFW) enriched with hydrogen or oxygen. Hydrogen-rich water (UF+H_2_) was prepared using reverse osmosis (RO) water to obtain 1000 ppb H_2_ molecules, and oxygen-rich water (UF+O_2_) water was prepared using RO water to contain 10 mg/L O_2_. Hydrogen concentration was determined with a portable Dissolved Hydrogen Meter (Trustlex Co., Ltd., ENH-1000, Tokyo, Japan). Dissolved oxygen (DO) content was determined using a Dissolved Oxygen Meter (Lutron Co., Ltd., PDO-519, Taipei, Taiwan).

### 4.2. Plant Materials and Growth Conditions

Melon (*Cucumis melo* L.) seeds were provided by Known-you Seed Co., Ltd. (Kaohsiung, Taiwan). Melon lines 6792T-744 (M1), 6792T-LD (M2), 6792T-LQ (M3), and a popular and high-quality melon cultivar “Camilla” were used in this study. To test the effect of UFW (containing H_2_ and O_2_) on seed germination, a total of 40 melon seeds per line were imbibed in RO water and UFW overnight and sown on a wetted tissue paper in a 125 × 125 × 20 mm square Petri dish (SPL, Gyeonggi-do, Republic of Korea). Seed germination rates were recorded one day after sowing. Germination was defined as when the root length was over half the seed length. The germinated seeds were transferred into a #104 plug tray containing peat moss (Known-you Seed). Then, the plug seedling was transplanted into a plastic pot (7.5 cm width × 7.5 cm height) containing 140 mL peat moss. The melon seedlings varied from 10 to 21 per line and were raised in a greenhouse at a temperature of 24 ± 4 °C.

The commercial melon cultivar “Camilla” was used to evaluate the effect of UF+H2, UF+O_2_, and a tap water control on the growth and fruit production in a plastic greenhouse of the Biotechnology Center in Southern Taiwan (AS-BCST) (23°06′14.4″ N 120°17′31.2″ E). We transplanted two melon seedlings at the four-leaf stage into a package of 80 L peat moss (Known-you Seed). A total of 22 seedlings were planted for each treatment. In the plastic greenhouse, we used tap water to prepare hydrogen-rich water (UF+H_2_) and oxygen-rich water (UF+O_2_), and the control was ordinary tap water. Water was supplied using a drip irrigation system. In this study, melons were grown vertically to keep the fruit cleaner and healthier. Flowers were pollinated at 13 to 16 nodes, retaining one fruit per plant. When the number of nodes on the mother vine reached 26, we removed the top growth point. According to the weather and soil moisture conditions, we supplied the appropriate water amount (500 mL~1000 mL per day per plant) during planting to ensure good plant growth and avoid fruit cracking in the later stages of fruit maturity, which may reduce fruit quality.

### 4.3. Insect Materials

Cotton aphids were collected from melon plants in a field of Tainan, Taiwan (23°14′56.0″ N 120°19′32.5″ E). The insects were reared in Camilla melon pot plants and placed in an insect cage. The cage was placed in a greenhouse at a temperature of 24 ± 4 °C. M1, M2, M3, and Camilla melon pot seedlings at four leave stages, of which each genotype has 10 to 21 seedlings, were placed in a walk-in growth chamber (25 ± 2 °C, 12 h photoperiod). Four aphids were released onto each melon plant. Each treatment has more than ten plants. The pot seedlings were irrigated with RO water and UFW, respectively. We assessed aphid population development at 14 days after infestation, and the number of aphids was rated per plant. A rating of 0 indicates no aphids were observed, and 9 indicates a high aphid density.

### 4.4. Observation of Trichome Density

To quantify trichome number, we collected the newly established leaves (L1) from 3 to 6 plants of the melon ‘Camilla’, excised the middle part of the petioles, and obtained images of trichomes under a dissecting microscope (Leica S9D, Hamburg, Germany) at 10× magnification. We counted the number of trichomes and calculated the average number of trichomes per square centimeter.

### 4.5. Cryo-Scanning Electron Microscopy

The first newly developed melon leaves (L1) were used to observe trichome development and aphid infestation on melon seedlings. The abaxial sides of the leaves were observed via cryogenic scanning electron microscopy (cryo-SEM), using a FEI Quanta 200/Quorum PP2000TR FEI 2007 high-resolution SEM at the Plant Cell Biology Core Laboratory in the Institute of Plant and Microbial Biology, Academia Sinica, Taipei, Taiwan. Briefly, leaf section samples containing insect tarsals were loaded onto frozen specimen holders and cryo-fixed in slush nitrogen (−210 °C), then rapidly transferred to a cryo-unit in the frozen state. Samples were imaged by cryo-SEM at an accelerating voltage of 20 kV.

### 4.6. Total RNA Isolation and Real-Time PCR

Total RNAs of melon leaf tissues were isolated using the TRIzol Plus RNA purification kit (Thermo Fisher Scientific, San Jose, CA, USA), treated with DNase (Promega, Madison, WI, USA), and the first-strand cDNA was synthesized using an M-MLV Reverse Transcriptase cDNA synthesis kit (Promega). Quantitative real-time PCR (RT–qPCR) reactions were performed using 2× KAPA SYBR FAST master mix (KAPA Biosystems, Wilmington, MA, USA), and performed for 35 cycles in a volume of 15 μL using complementary DNA reverse transcribed from 2 ng of total RNA. The RT-qPCR was performed using a CFX96 Real-Time PCR detection system (Bio-Rad, Hercules, CA, USA) and quantification analysis was performed using the CFX Manager Software version 3.1 (Bio-Rad). *β-Actin* (MELO3C023264) and *ADP ribosylation factor 1* (*ADP*, MELO3C023630) were used as reference genes for normalization. The primers used in this study are listed in Supplementary Table S1. Each sample had three biological replicates.

### 4.7. Detected JA and Methyl-JA Content

Melon leaf samples were snap-frozen in liquid nitrogen and ground to a fine powder with a pestle and mortar. The powder (500 mg) was suspended in 2.5 mL of ice-cold 50% MeOH (−20 °C). The extracts were vortexed for 5 min and then centrifuged at 3 °C at 4000 rpm for 15 min. The supernatants were collected and the pellets were re-extracted using 500 µL ice-cold 50% MeOH (−20 °C). The supernatants were combined and applied to Sep-Pak Vac 3 mL C18 200 mg cartridges (Waters, Milford, CT, USA) for sample clean-up and concentration. The cartridges were conditioned with 2 mL of MeOH and equilibrated with 2 mL of water, then 3 mL of sample was applied to the C18 cartridge. The Solid Phase Extraction (SPE) cartridges were eluted with 1 mL of 100% acetonitrile to release the MeJA, followed by a 1 mL clean-up with MeOH. The eluates from the cartridges were filtered through 0.22 μm filters, transferred into chromatography vials, and detected using an ultra-performance liquid chromatography high-resolution tandem mass spectrometry (UPLC-HRMS/MS) (Thermo Fisher Scientific). UPLC separation was carried out on a BEH C18 column (2.5 × 100 mm, 1.7 µm, Waters) at a 0.3 mL/min flow rate. The column oven temperature was 40 °C. The gradient program was applied using 0.1% formic acid (FA) in water (phase A), and 0.1% FA in ACN (phase B). The sample injection volume was 20 µL.

### 4.8. SPAD Value and Photosynthesis Rate of Melon

A non-destructive portable chlorophyll (Chl) meter SPAD-502 Plus (Konica Minolta Optics, Osaka, Japan) was used to measure the chlorophyll content of the fourth newly established melon leaf (L4) at 45 days after pollination. To determine the photosynthesis rate, we used a Li-600 Porometer/Fluorometers meter (Li-COR, Lincoln, NE, USA) to measure stomatal conductance (gsw), electron transport rate (ETR), and the quantum yield of PSII calculated from fluorescence parameters (ΦPSII) of the L1 leaves at 23 days after pollination.

### 4.9. Statistical Analysis

Student’s *t*-test was used to compare the difference between CK and UF+H_2_ or UF+O_2_ treatment among different genotypes. *p* values of less than 5% were considered statistically significant.

## 5. Conclusions

This study shows that ultrafine hydrogen-rich water can significantly promote root development and increase the fruit yield and the sweetness of melons. As shown in the paper, we found that ultrafine water (UFW) significantly increased JA accumulation, increased *GL2* gene expression, and may induce trichome development to reduce insect infestation. UFW is easy to use. Farmers can incorporate UFW into their irrigation systems. This could be a promising non-pesticide crop protection method. It is beneficial to high-economic crops and organic farming. Farmers can work towards safer and more sustainable melon production systems. For basic research, the underlying molecular mechanisms of how ultrafine water promotes insect resistance deserve detailed investigation.

## Data Availability

Data supporting the findings of this study are available within this paper and its Supplementary Data published online.

## Supplementary Materials

The following supporting information can be downloaded at: https://www.mdpi.com/article/10.3390/plants13040537/s1, Supplementary Figure S1. Morphology of trichomes in different tissues of melon after ultrafine water irrigation; Supplementary Table S1. List of primers used in this study.
