# Supplementary material for: Safe Farming: Ultrafine Bubble Water Reduces Insect Infestation and Improves Melon Yield and Quality
Source: Plants (Basel). 2024 Feb 16;13(4):537. doi: 10.3390/plants13040537 (PMC10891724; doi:10.3390/plants13040537)
Supplement: Supplementary file 1 [file plants-13-00537-s001.zip › plants-2808894-supplementary.pdf]

Supplementary data:

Supplementary Table S1. List of primers used in this study.

Gene	Forward primer	Reverse primer	Accession No.
β-Actin	CCTGGTATCGCTGACCGTAT	TACTGAGCGATGCAAGGATG	MELO3C023264
ADP	ATATTGCCAACAAGGCGTAGA	TGCCCGTAAACAAGGGATAAA	MELO3C023630
GL2	TTAATCAATAATCCCACCGCC	CGGCTCAGAGTTATCACTACAGCT	MELO3C024256
JAZ	CAGCCCTCAAAGCCAACAAC	CTCTGAGGTCTCGCTTTCG	MELO3C006046
JMT	GGGCCCAACACGTTACTTCT	AAGACGTCGATGGAACTCGG	MELO3C003803

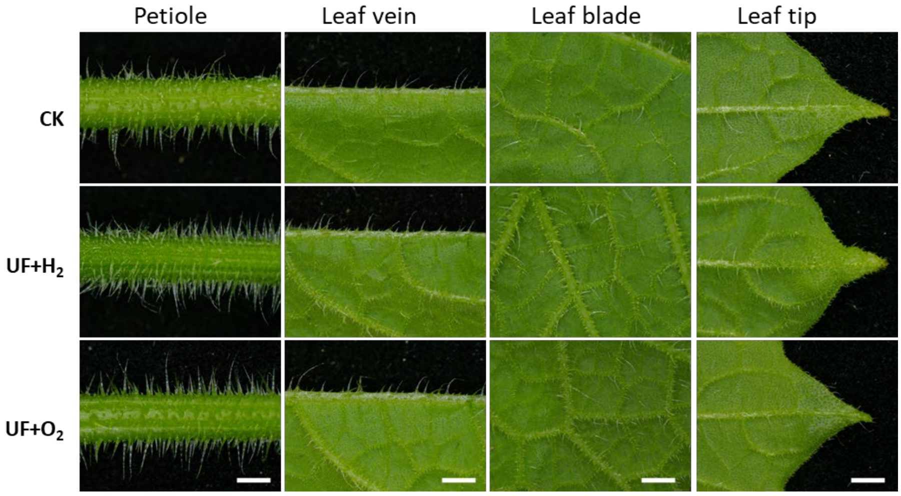

Figure S1. Morphology of trichomes in different tissues of melon after ultrafine water irrigation. The morphology of trichome development after melons were irrigated with ultrafine water enrichment of hydrogen (UF+H_2), oxygen (UF+O_2), and RO water control. Scale bar, 2 mm.
